# Low Expression in *Xenopus* Oocytes and Unusual Functional Properties of α_1_β_2_γ_2_ GABA_A_ Receptors with Non-Conventional Subunit Arrangement

**DOI:** 10.1371/journal.pone.0170572

**Published:** 2017-01-23

**Authors:** Roland Baur, Erwin Sigel

**Affiliations:** Institute of Biochemistry and Molecular Medicine, University of Bern, Bern, Switzerland; Weizmann Institute of Science, ISRAEL

## Abstract

The major subunit isoform of GABA_A_ receptors is α_1_β_2_γ_2_. The subunits are thought to surround an ion pore with the counterclockwise arrangement α_1_γ_2_β_2_α_1_β_2_ as seen from the outside of the neuron. These receptors have two agonist sites and one high affinity drug binding site specific for benzodiazepines. Recently, this receptor was postulated to assume alternative subunit stoichiometries and arrangements resulting in only one agonist site and one or even two sites for benzodiazepines. In order to force a defined subunit arrangement we expressed a combination of triple and dual concatenated subunits. Here we report that these unconventional receptors express only small current amplitudes in *Xenopus* oocytes. We determined agonist properties and modulation by diazepam of two of these receptors that resulted in currents large enough for a characterization, that is, β_2_-α_1_-γ_2_/α_1_-γ_2_ and β_2_-α_1_-γ_2_/β_2_-γ_2_. The first pentamer predicted to have two benzodiazepine binding sites shows similar response to diazepam as the standard receptor. As expected for both receptors with a single predicted agonist site the concentration response curves for GABA were characterized by a Hill coefficient < 1. β_2_-α_1_-γ_2_/β_2_-γ_2_ displayed a mM apparent GABA affinity for channel opening instead of the expected μM affinity. Based on their subunit and binding site stoichiometry, that contradicts all previous observations, their unusual functional properties and their very low expression levels in oocytes, we consider it unlikely that these unconventional receptors are expressed in neurons to an appreciable extent.

## Introduction

GABA_A_ receptors are the major inhibitory neurotransmitter receptors in mammalian brain [1–3]. The major isoform of this receptor is composed of α_1_, β_2_ and γ_2_ subunits. The consensus subunit stoichiometry is 2α_1_, 2β_2_ and 1γ_2_ [4,5]_,_ arranged α_1_γ2β2α1β2 counterclockwise as seen from the synaptic cleft [6–9]. α_1_β_2_γ_2_ receptors have one benzodiazepine binding site and two GABA sites [10], located at the α_1/_γ_2_ and β_2_/α_1_ subunit interfaces, respectively in a homologous position [3]. The presence of more than one agonist site for GABA is also predicted from their GABA concentration dependence that are characterized by a Hill coefficient of >1 (e.g. [8]). These α_1_β_2_γ_2_ receptors, unlike many other isoform containing δ or ε subunits [11–15], are thought to have a well-defined subunit arrangement. Subunit stoichiometry and arrangement are important as they govern functional properties [16]. Recently, unconventional subunit arrangements of α_1_β_2_γ_2_ receptors have been proposed [17]. Some of these receptors do not conform to the above stoichiometry as they contain 2γ_2_ subunits. Furthermore, these receptors are predicted to have one agonist site only and some of them two benzodiazepine sites. Functional properties of these novel receptors, such as EC_50_ and corresponding Hill coefficient for the agonist GABA and modulation by diazepam were not analyzed. We set out to determine these properties. While receptors conforming the conventional subunit arrangement could easily expressed in *Xenopus* oocytes, the receptors with unconventional arrangement form rather inefficiently, making their analysis difficult. Nevertheless, we describe the properties of some of them, but think that if these receptors are formed at all, they represent only a very minor part of α_1_β_2_γ_2_ GABA_A_ receptors.

## Materials and Methods

### Expression of GABA_A_ receptors in xenopus oocytes

Capped cRNAs were synthesized (Ambion, Austin, TX, USA) from the linearized plasmids with a cytomegalovirus promotor (pCMV vectors) containing the different subunits, respectively. A poly-A tail of about 400 residues was added to each transcript using yeast poly-A polymerase (United States Biologicals, Cleveland, OH, USA). The concentration of the cRNA was quantified on a formaldehyde gel using Radiant Red stain (Bio-Rad) for visualization of the RNA. Known concentrations of RNA ladder (Invitrogen) were loaded as standard on the same gel. cRNAs were precipitated in ethanol/isoamylalcohol 19:1, the dried pellet dissolved in water and stored at −80°C. cRNA mixtures were prepared from these stock solutions and stored at −80°C.

Animal experiments were carried out in strict accordance to the Swiss ethical guidelines, and have been approved by the local committee of the Canton Bern Kantonstierarzt, Kantonaler Veterinärdienst Bern (BE85/15). Surgery of female adult *Xenopus laevis* was done under anesthesia (0.2% tricaine solution). Oocytes were prepared, injected and defolliculated as described previously [18]. cRNA coding for each dual and triple subunit concatemer was injected either alone or in different combinations in oocytes. Oocytes were injected with 50 nl RNA solution containing RNA. In the case of non-concatenated α1β2γ_2_ receptors, cRNA coding for α_1_, β2 and γ_2_ subunits were injected at a ratio of 0.5:0.5:2.5 fMol/oocyte. In case of concatenated receptors, oocytes were injected with cRNA coding for dual and triple subunits at 1.25 fMol each. Injected oocytes were incubated in modified Barth’s solution at 18°C for at least 24 h before the measurements.

### Functional characterization of the GABA_A_ receptors

We used a two-electrode voltage clamp amplifier in combination with a XY-recorder (90% response time 0.1 s) or digitized at 100 Hz using a PowerLab 2/20 using the computer programs Chart (ADInstruments–Europe, Oxford, England). Electrodes were filled with 3 M KCl and had resistances of 0.5–0.8 MΩ. Tests with a model oocyte were performed to ensure linearity in the larger current range. The response was linear up to 15 μA. The holding membrane potential was −80 mV. The perfusion medium contained 90 mM NaCl, 1 mM KCl, 1 mM MgCl_2_, 1 mM CaCl_2_, and 5 mM Na-HEPES (pH 7.4). Concentration response curves for the compounds were fitted with the equation I(c) = I_max_/[1 + (EC_50_/c)^n^], where c is the concentration of the compound, EC_50_ the concentration eliciting half-maximal current amplitude, I_max_ is the maximal current amplitude, I the current amplitude, and n is the Hill coefficient. Maximal current amplitudes (I_max_) were obtained from the fits of the concentration-response curves. For all receptors studied, modulation was measured at a GABA concentration eliciting 1–3% of the maximal GABA current amplitude. GABA was applied twice alone, and 30 s application in combination with the different compounds. The duration of washout periods was 3 min in between agonist or agonist/drug aplications to prevent receptor desensitization. At the beginning of the experiments, GABA applications were repeated when the elicited current amplitude altered by >5%. Potentiation was calculated by the following equation: (I_Modulator + GABA_/I_GABA_− 1) * 100%. The perfusion solution was applied through a glass capillary with an inner diameter of 1.35 mm, the mouth of which was placed about 0.4 mm from the surface of the oocyte. This allowed fast changes in agonist concentration around the oocyte. The rate of change was estimated 70% in less than 0.5 s. The perfusion system was cleaned between drug applications by washing with DMSO to avoid contamination.

All data are from as a minimum of three different oocytes from at least two different batches of oocytes. Data represent mean ± SEM or SD as indicated.

## Results and Discussion

**Fig 1:**
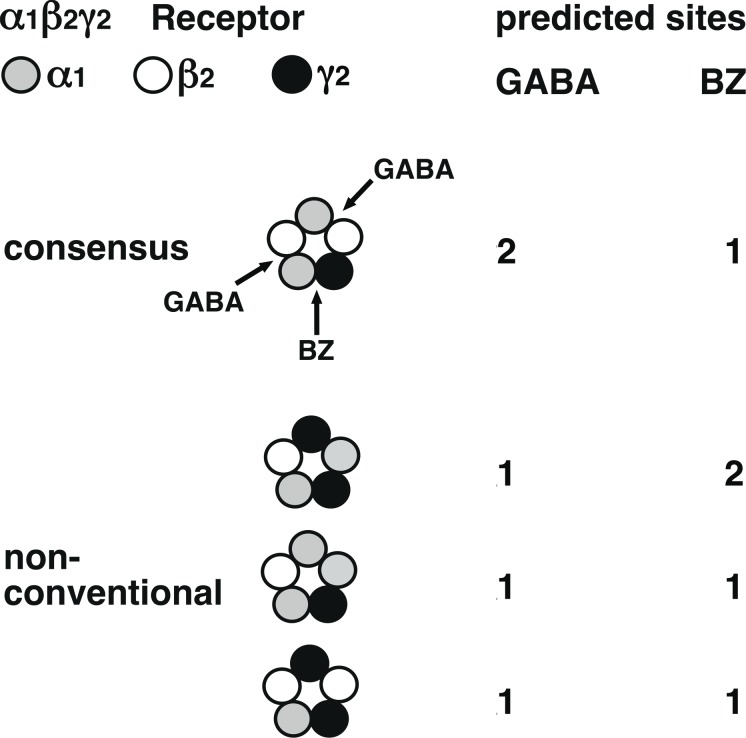
Consensus subunit arrangement of α_1_β_2_γ_2_ GABA_A_ receptors and three newly proposed receptors with unconventional subunit arrangement. Number of predicted agonist sites for GABA and modulatory sites for benzodiazepines (BZ) is shown for each receptor form. On the top left is the color code for the subunits.

The subunit arrangement of the α_1_β_2_γ_2_ GABA_A_ receptor has been derived from subunit interface studies [6] and from work with concatenated subunits [7–9]. Fig 1 shows a this arrangement characterized by the presence of two β_2_/α_1_ subunit interfaces each harboring agonist sites for GABA and one α_1_/γ_2_ subunit interface harboring a benzodiazepine binding site. This is in agreement with the observation that receptors purified from bovine brain have more than one GABA site per benzodiazepine binding site [10]. GABA concentration response curves obtained for α_1_β_2_γ_2_ GABA_A_ receptors also indicate the presence of more than one agonist site (e.g. [19]), as their Hill coefficient is > 1. In addition to the consensus receptor, Fig 1 shows three unconventional receptors [17] with one predicted agonist site each, two of them with one, one of them with two predicted benzodiazepine binding sites. All these unconventional receptors do not conform to the well-established subunit stoichiometry of 2:2:1 for α_1_, β_2_ and γ_2_ subunits [4,5]. We attempted to characterize the functional properties of these unconventional receptors.

**Fig 2:**
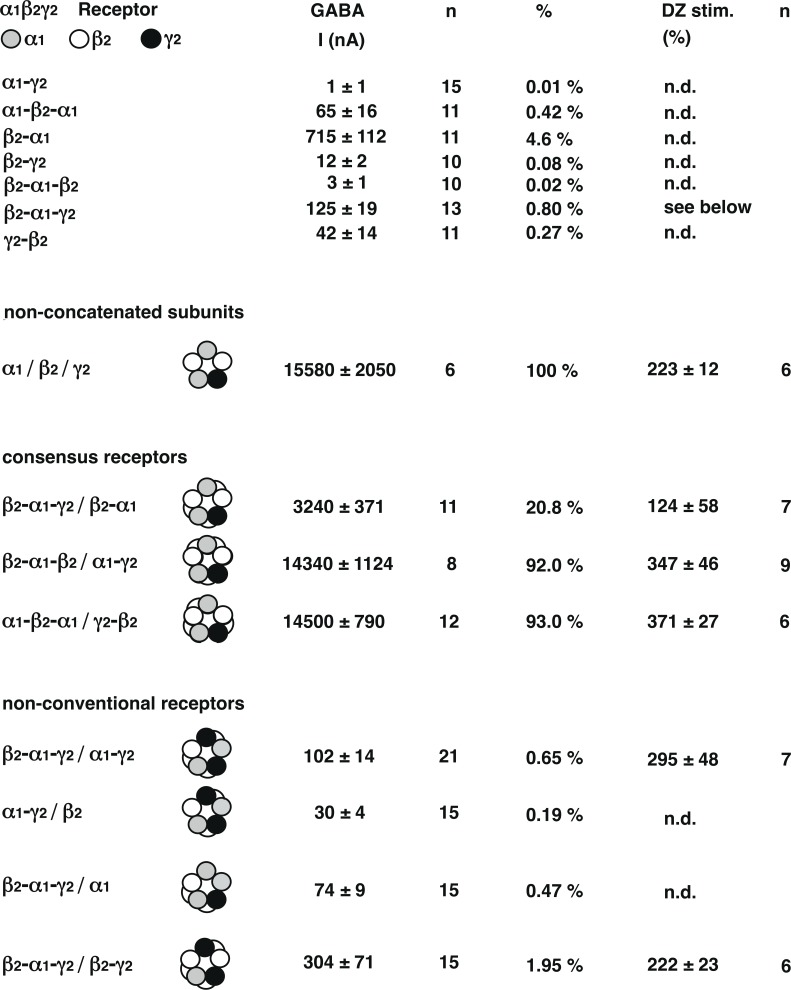
Dual and triple concatenated constructs used for the construction of pentameric receptors were expressed in *Xenopus* oocytes. Currents were standardized to the amplitude elicited in oocytes expressing non-concatenated α_1_, β_2_ and γ_2_ subunits (non-concatenated receptors). The same parameters are shown for receptors with consensus subunit arrangement (consensus receptors) that were built in three different ways and for three receptors with non-consensus subunit arrangement (non-consensus receptors), one of them built in two different ways. For some receptors, stimulation by 1 μM diazepam is also shown.

Non-concatenated α_1_β_2_γ_2_ GABA_A_ receptors were expressed in *Xenopus* oocytes. Current amplitudes elicited by 1 mM GABA were determined as about 15 μA. This current amplitude was compared with those resulting from three concatenated dual subunit constructs and three triple subunit constructs used later as part of pentameric receptors (Fig 2). While α_1_-γ_2_, β_2_-γ_2_, γ_2_-β_2_, α_1_-β_2_-α_1_ and β_2_-α_1_-β_2_ all resulted in the relative expression of less than 0.5% of the above current amplitude, β_2_-α_1_ and β_2_-α_1_-γ_2_ resulted in 4.6% and 0.8%, respectively. This extent of current expression from single concatenated subunits was not observed in previous work [7]. The reason for the discrepancy is not clear.

Non-concatenated receptors were also compared with three concatenated receptors with the consensus subunit arrangement and with four concatenated receptors with unusual subunit arrangement (Fig 2). Data on β_2_-α_1_-γ_2_/β_2_-α_1_ should be taken with care as the individual concatenated subunits result in current expression. All receptors with unusual subunit arrangement formed very inefficiently with expression levels well below 1%, with the exception of β_2_-α_1_-γ_2_/β_2_-γ_2_ with 1.95% (Fig 2). For two of these receptors, namely β_2_-α_1_-γ_2_/α_1_ and α_1_-γ_2_/β_2_, currents were too small for a further analysis. The observation that β_2_-α_1_-γ_2_/α_1_ fails to express has been made before [7]. In control experiments, β_2_-α_1_-γ_2_/α_1_ was expressed at a RNA ratio of 1:2. The current amplitude elicited by 1 mM GABA amounted to 108 ± 13 nA (mean ± SEM, n = 5). This is not significantly different from the same receptors expressed at an 1:1 ratio (Fig 2).

**Fig 3:**
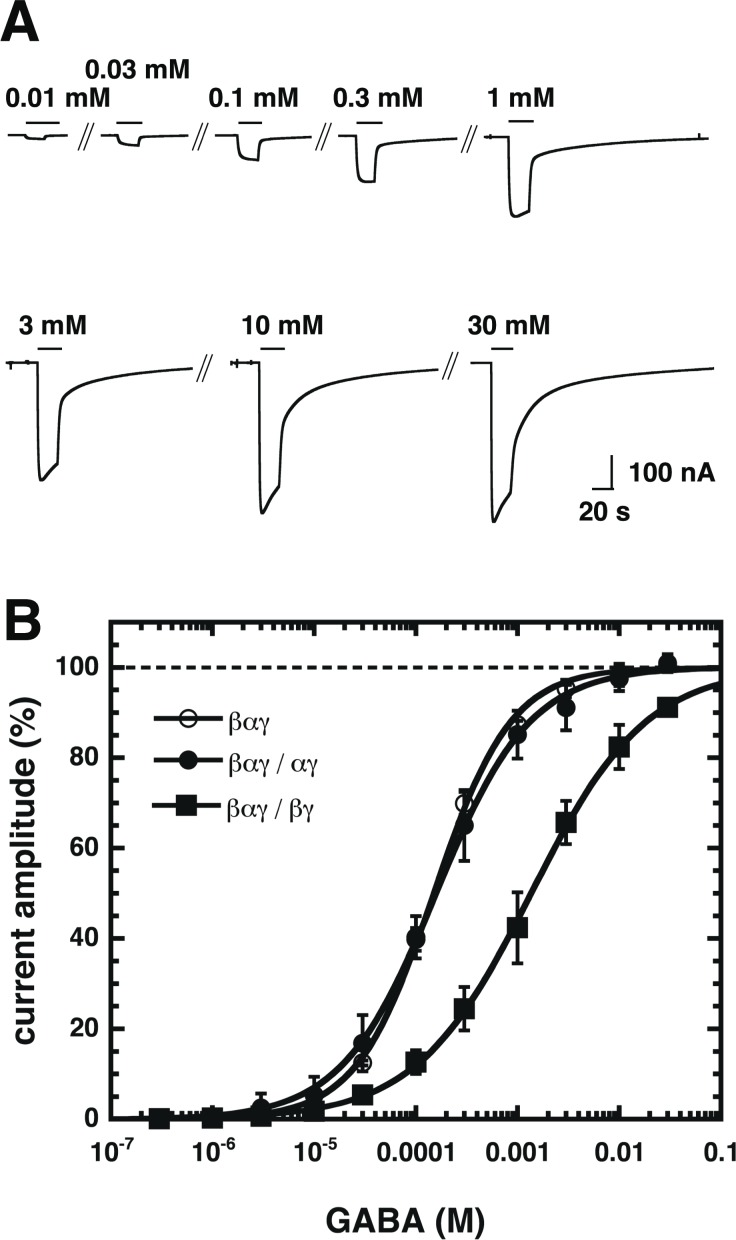
Concentration-response curves for β_2_-α_1_-γ_2_, β_2_-α_1_-γ_2_/α_1_-γ_2_ and β_2_-α_1_-γ_2_/β_2_-γ_2_ receptors. Receptors were exposed to subsequently higher concentrations of GABA and the elicited current amplitude was determined. Individual curves were first normalized to the fitted maximal current amplitude and subsequently averaged. Data are expressed as mean ± S.D., n = 3–5 from two batches of oocytes. A) Original current traces recorded in an oocyte expressing β_2_-α_1_-γ_2_/β_2_-γ_2_. B) Averaged Concentration-response curves for β_2_-α_1_-γ_2_, β_2_-α_1_-γ_2_/α_1_-γ_2_ and β_2_-α_1_-γ_2_/β_2_-γ_2_.

Agonist concentration-response curves were created for the non-conventional β_2_-α_1_-γ_2_/α_1_-γ_2_ and β_2_-α_1_-γ_2_/β_2_-γ_2_ receptors and for the triple construct β_2_-α_1_-γ_2_. Both pentameric receptors are predicted to have only one agonist site (Fig 1). Fig 3A shows original current traces for β_2_-α_1_-γ_2_/β_2_-γ_2_. After wash-out of GABA the current decreased in a bi-phasic manner. This unusual kinetic behavior was not observed with β_2_-α_1_-γ_2_ and β_2_-α_1_-γ_2_/α_1_-γ_2_. Fig 3B shows averaged concentration-response curves for β_2_-α_1_-γ_2_, β_2_-α_1_-γ_2_/α_1_-γ_2_ and β_2_-α_1_-γ_2_/β_2_-γ_2_ receptors. EC_50s_ and Hill coefficients characterizing the corresponding curves are shown in Table 1. Parameters describing the concentration response curve of β_2_-α_1_-γ_2_/α_1_-γ_2_ indicate efficient isomerization to the open state of the singly ligated receptor. The concentration dependence of β_2_-α_1_-γ_2_/β_2_-γ_2_ receptors differs strongly from receptors built from non-concatenated α_1_, β_2_ and γ_2_ subunits with an EC_50_ of about 40 μM and a Hill coefficient of about 1.4 [8]. This indicates that little if any β_2_-α_1_-γ_2_/β_2_-γ_2_ receptors are built from non-concatenated α_1_, β_2_ and γ_2_ subunits.

**Table 1:** Properties of GABA concentration response curves.

receptor	EC_50_ (μM)	Hill coeff.	n
β_2_-α_1_-γ_2_	142 ± 18	1.14 ± 0.03	3
β_2_-α_1_-γ_2_/α_1_-γ_2_	163 ± 35	0.97 ± 0.23	5
β_2_-α_1_-γ_2_/β_2_-γ_2_	1420 ± 530	0.78 ± 0.02	3

EC_50s_ are given as mean ± SD.

The fact that amplitude and agonist dependent properties of currents resulting from β_2_-α_1_-γ_2_/α_1_-γ_2_ are similar to the ones resulting from β_2_-α_1_-γ_2_ could suggest that two copies of β_2_-α_1_-γ_2_ form a pentameric receptor with one β_2_ hanging out, such that the pentamer would be β_2_-α_1_-γ_2_/α_1_-γ_2_. However, the Hill coefficient characterizing the concentration response curve for GABA at β_2_-α_1_-γ_2_ indicates that at least in some receptors the γ_2_ subunit must hang out, to allow formation of a second agonist site.

Modulation of the currents by 1 μM diazepam was determined. As reported before [8] concatenated receptors with consensus subunit arrangement showed a larger stimulation than non-concatenated receptors (Fig 2). Presumably, injection of α_1_, β_2_ and γ_2_ subunits even at a ratio of 1:1:5 results in diazepam responsive α_1_β_2_γ_2_ receptors and diazepam non-responsive α_1_β_2_ receptors. As expected, receptors composed of concatenated β_2_-α_1_ and β_2_-α_1_-γ_2_ showed reduced extent of modulation, as the current may partly be produced by the individual concatenated constructs, specifically by β_2_-α_1_.

**Fig 4:**
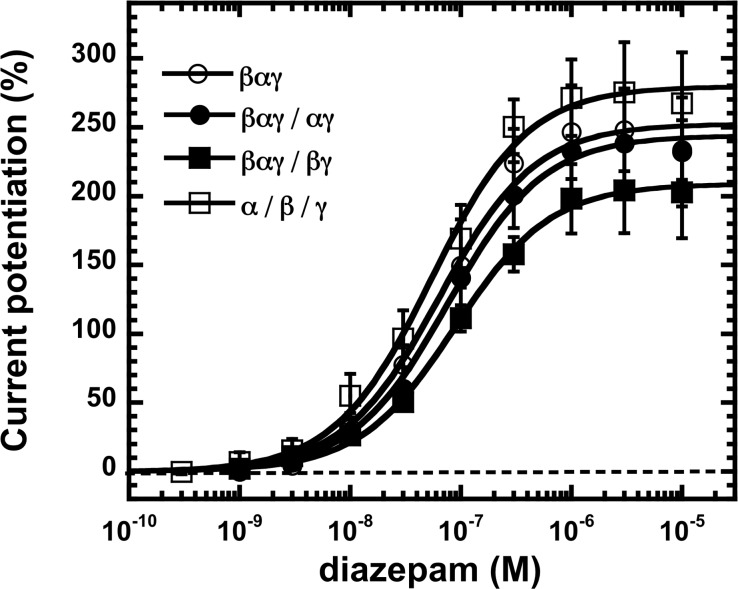
Stimulation of β_2_-α_1_-γ_2_, β_2_-α_1_-γ_2_/α_1_-γ_2_, β_2_-α_1_-γ_2_/β_2_-γ_2_ and α_1_/β_2_/γ_2_ receptors by diazepam. Receptors were exposed to subsequently higher concentrations of diazepam in combination with an EC_2_ GABA concentration and the elicited current amplitude was determined. Individual curves were first normalized to the fitted maximal current amplitude and subsequently averaged. Data are expressed as mean ± S.D., n = 3 from two batches of oocytes.

Effects of diazepam at the non-conventional β_2_-α_1_-γ_2_/α_1_-γ_2_, α_1_-γ_2_/β_2_ and β_2_-α_1_-γ_2_/β_2_-γ_2_ receptors were also determined. The first two receptors are predicted to have two sites for benzodiazepines. 1 μM diazepam by itself did not elicit any currents in β_2_-α_1_-γ_2_/α_1_-γ_2_ receptors (n = 5) and α_1_-γ_2_/β_2_ receptors (n = 3). Modulation of GABA current by the same concentration of diazepam in β_2_-α_1_-γ_2_/α_1_-γ_2_ receptors was comparable to that in concatenated consensus receptors, in spite of the predicted presence of two benzodiazepine binding sites in a receptor. In β_2_-α_1_-γ_2_/β_2_-γ_2_ receptors extent of modulation was slightly lower (Fig 2). Fig 4 shows averaged diazepam concentration-response curves from oocytes expressing β_2_-α_1_-γ_2_, β_2_-α_1_-γ_2_/α_1_-γ_2_, β_2_-α_1_-γ_2_/β_2_-γ_2_ or α_1_/β_2_/γ_2_ receptors. The curves were characterized by EC_50_s of 65 ± 9 nM (mean ± SD, n = 3), 77 ± 20 nM (mean ± SD, n = 3), 88 ± 18 nM (mean ± SD, n = 3) and 59 ± 13 nM (mean ± SD, n = 3), respectively. Maximal current stimulation was similar for all constructs varying between 209 ± 31% for β_2_-α_1_-γ_2_/ β_2_-γ_2_ and 285 ± 29% for α_1_/β_2_/γ_2_. The fact that the curve for β_2_-α_1_-γ_2_/α_1_-γ_2_ looks similar to that for α_1_/β_2_/γ_2_ [8] indicates that the two predicted sites show no cooperativity or additivity of modulatory effects.

Concerning receptor subunit stoichiometry, the subset of GABA_A_ receptors containing binding site for benzodiazepines, mainly α_1_β_x_γ_2_, α_2_β_x_γ_2_, α_3_β_x_γ_2_ and α_5_β_x_γ_2,_ purified from bovine brain using a benzodiazepine affinity column [10] show a stoichiometry of agonist sites to benzodiazepine binding sites of 1.2–2.4. The stoichiometry of non-conventional GABA_A_ receptors β_2_α_1_γ_2_α_1_α_1_, β_2_α_1_γ_2_α_1_γ_2_ and β_2_α_1_γ_2_β_2_γ_2_ would be predicted to be 1:1, 0.5:1 and 1:1, respectively [17] and that of conventional GABA_A_ receptors 2:1. The subunit stoichiometries of non-conventional GABA_A_ receptors clearly do not agree to the reported stoichiometry.

In summary, the non-conventional α_1_β_2_γ_2_ GABA_A_ receptors are not in agreement with literature data on subunit and binding site stoichiometry and functional properties. They show low expression levels in *Xenopus* oocytes. Functional properties, e.g very low GABA affinity for β_2_-α_1_-γ_2_/β_2_-γ_2_ channel gating and low Hill coefficient for β_2_-α_1_-γ_2_/α_1_-γ_2_ differ from those of non-concatenated α_1_β_2_γ_2_ GABA_A_ receptors expressed in various expression systems. This together makes it very unlikely that non-conventional GABA_A_ receptors of the subunit arrangement β_2_α_1_γ_2_α_1_α_1_, β_2_α_1_γ_2_α_1_γ_2_ and β_2_α_1_γ_2_β_2_γ_2_ are being formed to any appreciable extent. Non-concatenated α_1_, β_2_ and γ_2_ subunits as previously concluded most likely assemble to β_2_α_1_γ_2_β_2_α_1_ GABA_A_ receptors.
